# Notes and Notices

**Published:** 1897-01

**Authors:** 


### NOTES AND NOTICES.

Married.—Dr. Maro F. Underwood and Miss Anna C. Lawler were married on New Year's Eve, 1896. Their address will hereafter be 1302 West Street, San Francisco, Cal.

Dr. John Storer wishes to announce that, in connection with his general practice, he is now prepared to treat, as a specialist, all diseases of the eye and ear, including the proper fitting of glasses. Office, 16 Revere Street, Jamaica Plain. Hours: 8 to 9 a. m.; 1 to 2.30, and 6.30 to 7.30 p. m. Sundays, 12 to 1 p. m.

Stamford Hall.—At Stamford, Conn., Dr. Amos J. Givens has a well-appointed sanitarium for the treatment of nervous and mild mental diseases, with separate apartments for narcotic and alcoholic habitues.

Dr. Givens is a well-known member of our profession, and physicians may, with the utmost confidence, place their patients in his care.

The Observer.—Increasing and pressing financial troubles due to the long business depression compelled E. F. Bigelow to make an assignment, October 9th, 1896, to Edward H. Wilkins, of Portland. This meant that available resources were not in hand or could not be collected to meet all over-due and urgently demanded bills, necessitating a surrender, as the law provides, of all personal and real estate into the hands of a trustee to dispose of for the benefit of the creditors. You may understand, theoretically, what it means to have taken away all the accumulations of years of hard work, except the few things exempted by law, but no one who has not been through it can actually understand and realize it.

Though the principal part of the causes resulting in this assignment were not in *The Observer*, but the allied branches of a large printing and publishing business, yet *The Observer*, of course, was carried down with the rest and its publication discontinued. This left all advance payments on *Observer* subscriptions as liabilities, and so reported by Mr. Bigelow to the Probate Court in the list of liabilities at time of assignment. Each subscriber is a creditor entitled to hand in claim against the estate, same as any other creditor, for the amount due on unexpired advance-paid subscriptions. Notice to that effect was given by postal-card to each subscriber by the commissioners appointed to receive and examine claims against the estate.

On December 18th, 1896, the good-will, outfit, back numbers, stationery, etc., of *The Observer* were purchased of the trustee by Mrs. E. A. Pelton, of Portland, who is the mother of Mrs. E. F. Bigelow. Mrs. Pelton has appointed E. F. Bigelow as agent and managing editor of *The Observer* and the magazine will be continued practically as before, except with many plans and reasonable expectations of improvement for 1897. [*The Observer* is a scientific journal which has been frequently reviewed in these pages, during the past year. See March number, p. 153, July number, p. 336, and October number, p. 452.—Ed.]

The Funk & Wagnalls Company, of 30 Lafayette Place, New York, propose to send to any physician, free of charge, a great reference work of nearly one thousand large royal octavo pages, bound substantially and expressly in card-manilla paper, delivered free at his own door, on a condition so tempting that such is possible only, on their part, because of future business. This book is regularly catalogued and listed at $6.50, bound in tan sheep. Now, by manufacturing in very large quantities, they are able to use a first-class paper, printed with the same degree of accuracy and finish, and have the same kind of binder's work, as to folding, collating, sewing, etc., and, by using card-manilla paper as a cover, send the book to the physician without the cost of one penny, on the simple condition that he will agree to subscribe for a periodical that is especially valuable to him as a professional man, at a subscription price of *even less than one-half* the above-named price of the book, which is to be sent him. For particulars of this astonishing offer, write to Messrs. Funk & Wagnalls for a circular of information.

